# Noma in a boy with septic shock: a case report

**DOI:** 10.1186/s12887-019-1574-8

**Published:** 2019-06-17

**Authors:** Lili Xu, Wanrui Wei, Xiaohua Ge, Sibei Wan, Jing Yu, Xiaodong Zhu

**Affiliations:** 1 PICU, Xin-Hua Hospital Affiliated to Shanghai Jiao Tong University School of Medicine, Shanghai, China; 2 School of Nursing, Shanghai Jiao Tong University, Shanghai, China; 3 Department of Nursing, Xin-Hua Hospital Affiliated to Shanghai Jiao Tong University School of Medicine, Shanghai, China

**Keywords:** Noma, Cancrum oris, Septic shock, Continuous renal replacement therapy, Pediatrics

## Abstract

**Background:**

Noma is a rare disease, which is characterized by rapid progression and a high rate of mortality; however, relatively few cases of noma infection accompanied by septic shock in children have been described. Further, most health care professionals have no knowledge of this disease or of its clinical significance.

**Case presentation:**

Herein, we present a case report of a six-year-old male patient who was diagnosed with noma infection at a Chinese pediatric medical intensive care unit (PMICU), at which time, it was discovered that he had septic shock. Following treatment by continuous renal replacement therapy (CRRT) for septic shock arising from noma, the patient was in generally good condition, and the local wound was seen to be essentially healed five weeks post-admission.

**Conclusion:**

Noma is an opportunistic infectious disease condition. Treatment of the acute phase of noma predominantly focuses on controlling the infection and improving systemic conditions. In addition, CRRT could be considered as a treatment option for cases that present with noma accompanied by septic shock.

## Background

Noma, which is commonly known as cancrum oris, is an acutely progressive and necrotic disease that is induced by infections, and most commonly seen in malnourished and immuno-suppressed children [1]. Moreover, if the patient is in septic shock, the mortality rate can be as high as 50% or more [2], and if left untreated, mortality may be up to 85% [3].

There are few reports in the peer-reviewed literature of pediatric noma that is complicated by septic shock. Furthermore, the treatment of noma in the acute phase of progression mainly serves to improve the general condition of the patient and the quality of life. Clinical management of this condition thus includes wound cleaning and debridement, maintaining the balance of water and electrolytes through rehydration support, improving and sustaining the nutritional condition of the patient, supplementing the patient with trace elements (especially of vitamin A) and appropriate use of antibiotics [3–5].

This report describes a case of noma that was complicated by septic shock, in which treatment consisted of continuous renal replacement therapy (CRRT) at a pediatric medical intensive care unit (PMICU).

## Case presentation

On January 16, 2016, a six-year-old male presented with two days of swelling of the right maxillofacial region with fever and two hours of weakness. He was diagnosed with noma and septic shock, and was admitted to the PMICU at Xinhua Hospital of Shanghai Jiao Tong University in Shanghai, China. The patient is of Chinese Han nationality/ethnicity. He had access to a clean water source, no previous related disease, no weight loss, no history of direct contact with poultry and feces, good general nutritional status, was up to date on his immunizations (at the appropriate age according to national regulations), an absence of any known family history of immunodeficiencies, with unknown sanitation in the home and with unknown oral hygiene status.

The right side of the mouth and the maxillofacial area were swollen and tender 36 h before admission. Then, the swollen area gradually expanded to the entire region of the right maxillofacial tissues. Moreover, the local skin color developed to a darkened red, and his temperature was noted to be 39.4 °C. Two hours before admission, the patient lacked energy and developed significant weakness. One day after the onset of symptoms, the patient developed diarrhea, passing seven to eight loose stools per day.

Admission examination revealed the following: a weight of 23.0 kg (71.0% for 6-year-old boy), a height of 123 cm (85.4% for 6-year-old boy), and a BMI of 15.20. The patient was listless (mental status), had a temperature of 37.4 °C, a heart rate of 163 beats/min, a respiratory rate of 24 breaths/min, a blood pressure of 60/40 mmHg, and a SaO_2_ of 90%. Results of the laboratory examinations are shown in Table 1.

**Table 1:** Results of Laboratory Examinations

Type of test	Patient’s value	Normal range
white blood cell count	14.86 × 10^9^/L	(4–10) 10^9^/L
neutrophil ratio	94.5%	(50–70)%
level of C reactive protein	> 160 mg/L	<8 mg/L
procalcitonin	> 100 ng/ml	<0.5 ng/ml
glucose	2.2 mmol/L	3.6–6.1 mmol/L
alanine aminotransferase	76 u/L	21–72 u/L
aspartate aminotransferase	113 u/L	17–59 u/L
albumin	27.6 g/L	35–50 g/L
prothrombin time	24.3 s	9–13 s
activated partial thromboplastin time	55.6 s	26–39 s
fibrinogen degradation product	52.64 mg/L	0–5 mg/L
CMV-DNA amplification	< 1 * 10^3^ copy numbers/ml	NA
(1–3)-β-D Glucan, Fungus(G test)^a^	101 pg/ml	<10 pg/ml
endotoxin	< 5 pg/ml	< 10 pg/ml
Human Immunodeficiency Virus	negative	NA
cold agglutinin test	1:128	1:32
*mycoplasma pneumoniae* antibody	positive	NA

^a^ The G test measures the cell wall composition of the fungus

**Fig. 1:**
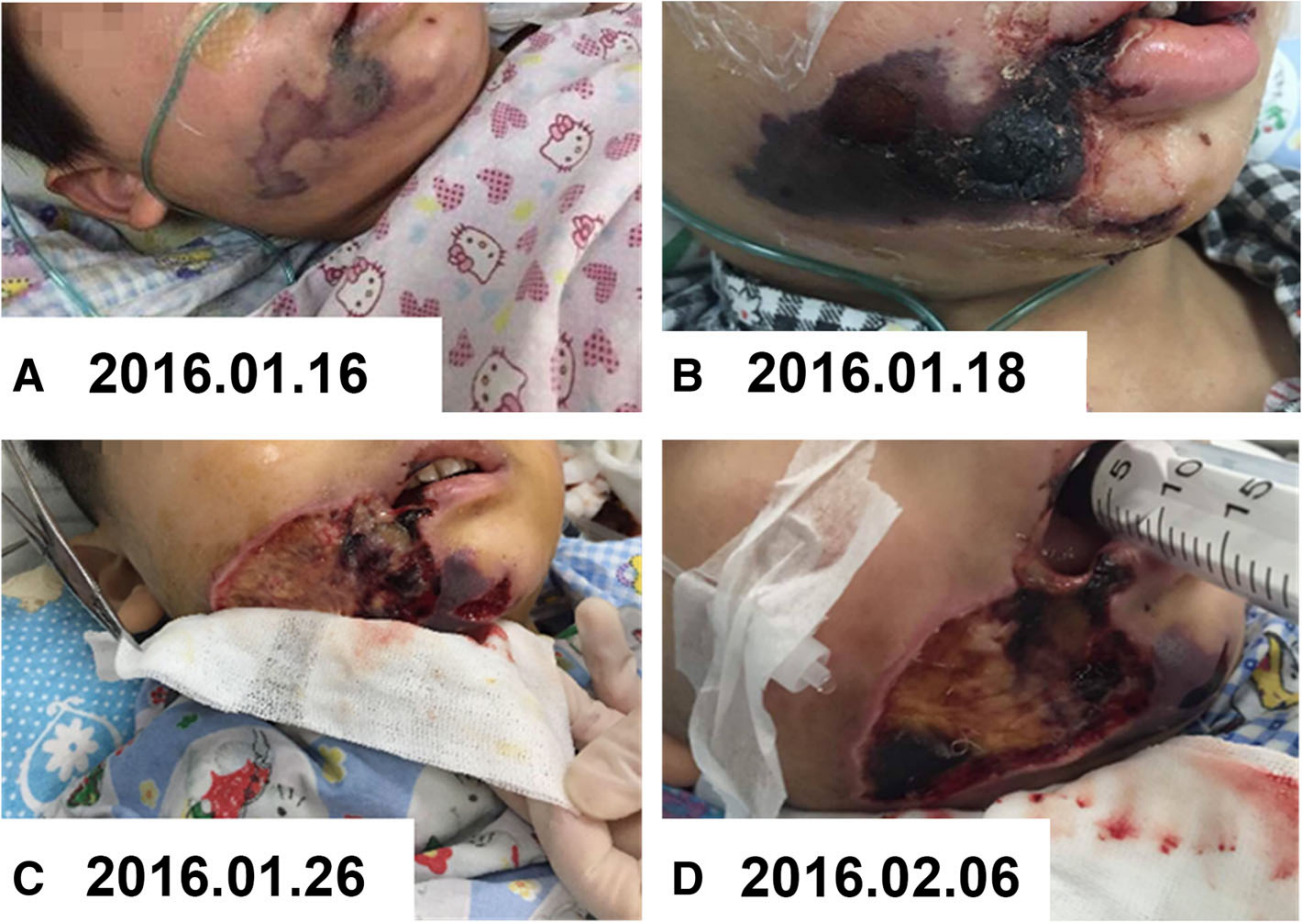
**a** The dark red ecchymosis of the local skin was seen to extend from the right sulcus to the lower forehead. **b** The ecchymoses had dispersed to the entire right cheek and parts of the lower jaw, following which, the skin and subcutaneous tissues appeared black and were hard to the touch. **c** The necrotic tissue under the clam-shaped zone was removed, exposing a large tissue defect of a triangular area in the right cheek and mandibular region. **d** Continued physical therapy that kept the mouth opening for two hours, for a frequency of 3-4 times each day

The swelling of the right cheek and an observation of local tenderness was obvious (3× 4 cm), and the skin temperature was increased without fluctuation. The dark red ecchymosis of the local skin was seen to extend from the right sulcus to the lower forehead (Fig. 1a). The oral mucosa was intact without ulceration, and there were many firm and non-adhesive enlarged lymph nodes on both sides of the neck. The skin of the extremities was cold, and the capillary refill time exceeded five seconds. Both lungs displayed thick and coarse breath sounds and wet rales.

After admission, the patient was given fluid resuscitation and anti-shock therapy with vasoactive drugs, including norepinephrine (0.05 mg/kg/min, 24-h maintenance), dopamine (5 mg/kg/min, 24-h maintenance); anti-infective treatment with linezolid (10 mg/kg/min, q8) and meropenem (20 mg/kg/min, q6); plasma albumin(10 mg/kg/min), gamma globulin (22.5 g/d); and CRRT (Swedish Campbell PRISMA blood purifier and M60 filter, dialysis method CVVHDF, and blood flow) was carried out. Blood flow was 70 ml/min, the speed of replacement fluid and dialysate was 550 ml/h, and the duration of dialysis was 12 h per day for three consecutive days. After six hours of treatment, the blood pressure of the patient gradually recovered (90/55 mmHg), and the heart rate decreased (130 beats/min). In addition, breathing was stable, and lung function began to improve. Thirty-six hours after admission, the vital signs of the patient stabilized and returned to normal levels, and vasoactive drug therapy was withdrawn.

On the first day, the swelling of the right cheek was obvious, and local skin ecchymoses of the cheeks gradually expanded. Two days after admission, the ecchymoses had dispersed to the entire right cheek and parts of the lower jaw, following which, the skin and subcutaneous tissues appeared black and were hard to the touch (Fig. 1b). The nurses made records of vital measurements and assessed the wounds on a daily basis. One week after admission, the skin and subcutaneous tissues had completely formed, and took the shape and a black colored clam-shaped shell (10 × 6 cm) attached to the right cheek, and the pain was reported to have disappeared. However, it should be noted that the pain duration could not be determined due to a lack of records as to when the pain began. Since the patient was too young to provide his opinions adequately, doctors obtained the perspectives and consent of the patient’s parents with regard all of these treatments.

Following treatment, the scar crust was surgically removed ten days after admission, and the necrotic tissue under the clam-shaped zone was removed, exposing a large tissue defect of a triangular area in the right cheek and mandibular region (Fig. 1c). During this period, nursing care was vital. The wound edge was cleaned daily with 0.9% sodium chloride solution and iodophor, and the wound was treated with applications of 10% sodium chloride solution continuously for 24 h. To ensure the wet dressing effect, the bed nurse used a 50 ml syringe with a micro-pump (4 ml/h) to extract 10% sodium chloride solution connected to an extension tube, with one end of the extension tube fixed to the center of the gauze that covered the wound. During treatment, the bed nurse checked the humidity of the wound gauze every hour to ensure no drip. In addition, the wound gauze was replaced every four hours. The bed nurse examined and cleared the necrotic subcutaneous tissue every 48 h, and checked wound healing progress at every shift.

**Fig. 2:**
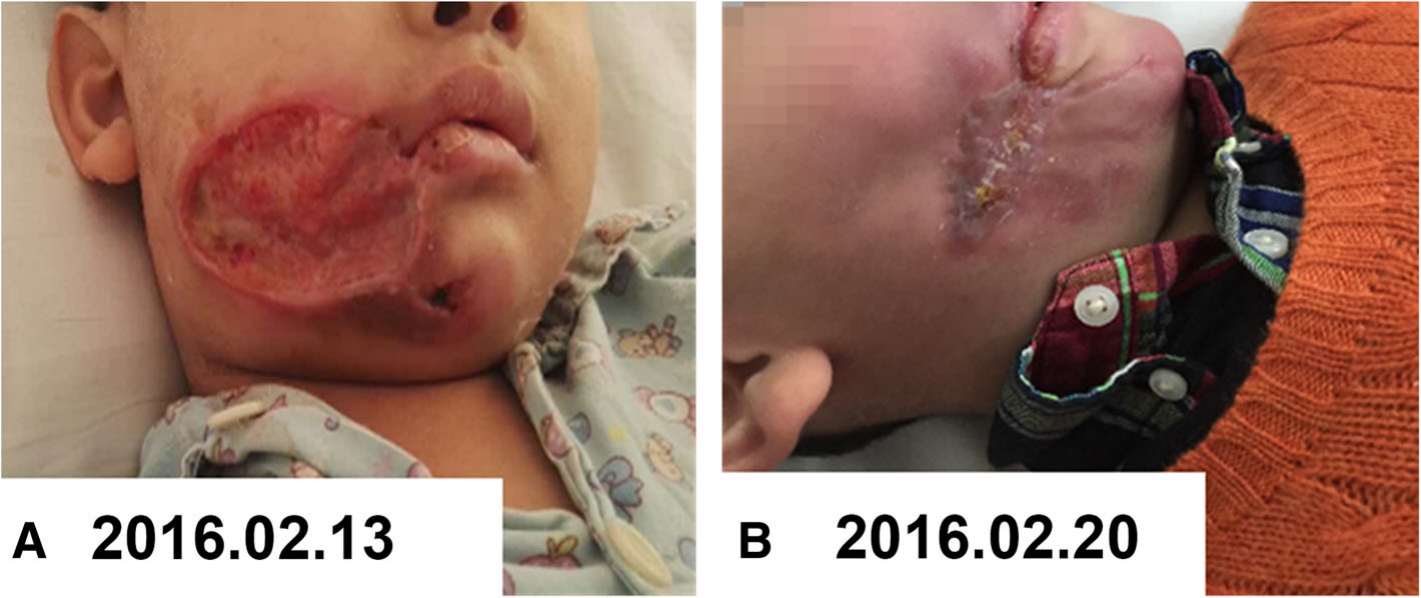
**a** The defective area of the right cheek and jaw was seen to be gradually reduced. **b** The local wound had essentially healed, and showed remarkable recovery

To reduce the restriction of the mouth opening because of fibrous scar contractions, the responsible nurse used a clean 20 ml syringe to replace gags (a device for keeping the patient’s mouth open during a dental or surgical operation) from the third week after admission, and continued physical therapy that kept the mouth opening for two hours, for a frequency of 3–4 times each day (Fig. 1d). On January 29, 2016, the patient was transferred to a medical department to continue anti-infective management, wound care, nutritional support and other symptomatic treatment options. Further, the defective area of the right cheek and jaw was seen to be gradually reduced (Fig. 2a), and the fibrous scar tissue had gradually been formed on the right cheek wound.

Five weeks after admission, the child was generally in good condition and the local wound had essentially healed, and showed remarkable recovery (Fig. 2b). The vital signs of the patient were stable and the patient was discharged on March 8, 2016. At that time, the right maxillofacial region was slightly swollen, and the wound defect was approximately 3 × 1 cm in dimension. The wound gauze was covered without exudation. The patient was discharged with a set therapeutic regimen that comprised the following: multi-vitamin tablets Sig: 0.5 tablets, oral, qd; ubiquinone capsules Sig: 10 mg, oral, q12h; and ursodeoxycholic acid tablets at 50 mg, oral, tid (ubiquinone capsules and ursodeoxycholic acid were used to treat slight liver dysfunction).

Two weeks after discharge, the patient visited the outpatient clinic of our hospital for follow-up, wherein the patient was shown to have recovered well, and it was recommended as standard practice that surgical cheek repair be performed. The exact time will be decided by the plastic rehabilitation surgeon in the plastic rehabilitation specialized hospital.

## Discussion

The cause of noma is unclear, and its main pathogenic factors might include malnutrition and low vaccination coverage [5], poor oral hygiene and home sanitation [6], pre-infection conditions like measles or diarrhea [7], and immunodeficiency [8], among other factors. The male patient in this report suffered from noma; however, the patient did not display any special contact or past history, and essentially presented with fever and diarrhea, which implied the patient might have suffered from a previous infection. Thus, the immediate cause of the disease remained uncertain. A related indicator of noma is severe poverty and an altered oral microbiota [4]. Rapid and effective control of severe sepsis and its complications is key to treating children with noma. We gave fluid resuscitation, anti-shock therapy and anti-infective therapy to the patient, as was done in other cases. Further, as CRRT has been used in treating severe sepsis [9, 10], we utilized CRRT after admission to reduce the systemic inflammatory response, control body temperature and regulate the acid-base balance.

In this current case, the patient arrived at the hospital after a short history of ineffective treatment at a local hospital, which included decreasing the extent of the fever (mellin ibuprofen suspension) and anti-infective therapy (piperacillin-tazobactam, metronidazole, ceftriaxone) in response to the fever and diarrhea. Then, it took two days for the patient to successfully arrive at our hospital, due to the treating doctors being unaware of the serious nature of noma. In another related case [11], a 9-year-old patient was treated one week after the onset of the condition - this situation was also severe. Therefore, it is vital to diagnose and treat this type of condition early in its course. Furthermore, only a few cases have been reported of noma with severe septic shock in developed countries, and the patients appear to always present with preceding infections (e.g., severe diarrhea, measles, malaria, and AIDS) [12] or chronic diseases with an associated immunodeficiency including the use of chronic steroids or other medications that impair immunity, such as in Crohn’s disease [13].

## Conclusion

If noma is not identified and treated in time, the mortality rate can be as high as 85%. Therefore, it is vital to access health care as quickly as possible by educating community health workers and the public on the early signs and symptoms of noma. Furthermore, improving an understanding and awareness of this preventable and treatable childhood disease by pediatric medical staff is critical to a successful early diagnosis and active treatment, which can ultimately reduce the harm from noma in the pediatric setting. Moreover, stakeholders should practice greater responsibility and specific action in recognizing and managing this adverse condition [3].

## Data Availability

The datasets used and/or analyzed during the current study are available from the corresponding author on reasonable request.

## Abbreviations

CRRT: Continuous renal replacement therapy
PMICU: Pediatric medical intensive care unit
